# Supplementary material for: Temporal and Spatial Dynamics of Organ Water Content in Maize with Different Senescence Types
Source: Plants (Basel). 2023 Sep 14;12(18):3269. doi: 10.3390/plants12183269 (PMC10535380; doi:10.3390/plants12183269)
Supplement: Supplementary file 1 [file plants-12-03269-s001.zip › plants-2582999-supplementary.pdf]

Supplementary materials

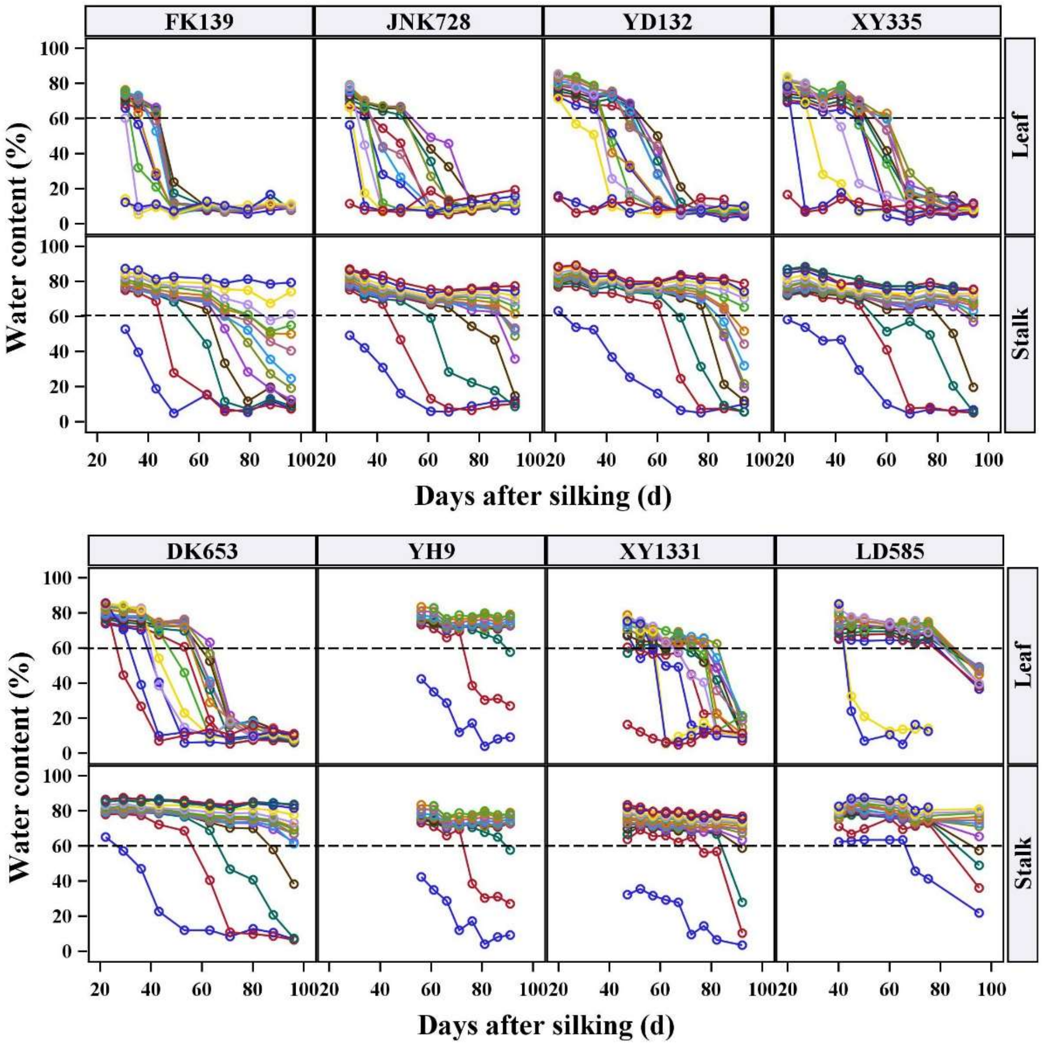

Supplementary Fig. S1 Relationship of days after silking and water dynamics of stalk internodes and leaves of four maize cultivars. The lines with different colored circles represented the changes of stalk internodes or leaves water content at different positions, and the same color in different pictures represented the internodes or leaves with same position. The dash line was the reference line of 60% water content. The maize cultivars names were: FK139 (Fengken139); JNK728 (Jingnongke728); XY335 (Xianyu335); YD132 (Yudan132); DK653 (Dika653); YH9 (Yuanhua9); XY1331 (Xianyu1331); LD585 (Liaodan585).

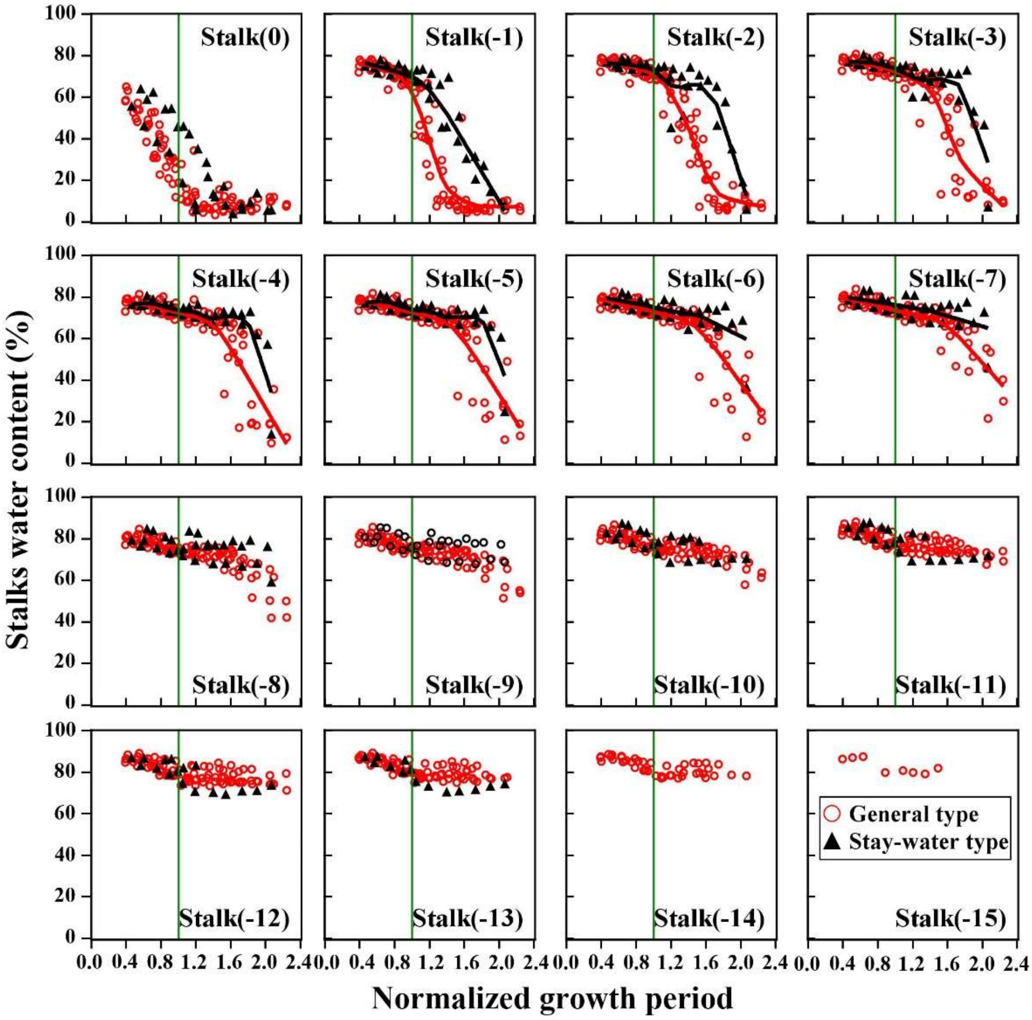

Supplementary Fig. S2 Differences of stalk internodes water content with different types at different positions. The colored lines represented the LOESS (Locally Weighted Regression) fitting lines of data

Supplementary Table S1 Frequency distribution of ear position in different maize cultivars

Cultivars	Ear frequency at different positions						Ear positions
	(-9)	(-8)	(-7)	(-6)	(-5)	(-4)	adopted
Fengken139			2/60	42/60	15/60	1/60	(-6)
Hetian1			1/60	20/60	38/60	1/60	(-5) and (-6)
Jingnongke728			4/60	51/60	2/60		(-6)
Dika517				27/60	33/60		(-5) and (-6)
Xianyu335			22/36	12/36	2/36		(-7)
Zhengdan958			1/60	43/60	16/60		(-6)
Zeyu8911		1/65	55/65	9/65			(-7)
Yudan132				19/36	16/36	1/36	(-5) and (-6)
Dika653			6/60	46/60	8/60		(-6)
Yuanhua9				2/30	16/30	12/30	(-4) and (-5)
Xianyu1331	1/30	8/30	21/30				(-7)
Liaodan585		17/27	10/27				(-7) and (-8)

Supplementary Table S2 Detail information of parameters of water dynamics equation of maize leaves in different types

Cultivars type	Parameters	Leaf Positions											
		(0)	(-1)	(-2)	(-3)	(-4)	(-5)	(-6)	(-7)	(-8)	(-9)	(-10)	(-11)
General type	k_1	-39.03	-23.81	-17.03	-16.02	-16.19	-20.99	-27.82	-31.97	-17.29	-20.79	-23.94	-21.14
	b_1	89.38	83.22	81.25	82.71	84.32	88.00	92.75	96.52	89.15	91.82	93.74	90.49
	k_2	-174.80	-221.70	-134.90	-118.70	-130.50	-141.40	-157.00	-142.50	-103.10	-119.10	-132.60	-135.80
	b_2	205.75	279.66	197.59	186.42	203.08	214.71	227.07	205.25	151.20	159.41	159.51	150.95
	b_0	7.21	8.15	9.70	11.72	11.88	11.11	11.09	10.80	10.04	10.55	11.16	10.65
	R^2	0.94**	0.95**	0.94**	0.92**	0.90**	0.92**	0.90**	0.92**	0.89**	0.88**	0.83**	0.79**
	F value	307.78	439.09	305.31	235.15	183.07	229.48	188.62	247.38	175.75	149.1	104.27	76.91
	P_1	0.86	0.99	0.99	1.01	1.04	1.05	1.04	0.98	0.72	0.69	0.61	0.53
	P_2	1.14	1.22	1.39	1.47	1.47	1.44	1.38	1.36	1.37	1.25	1.12	1.03
	Water content at P_1	55.93	59.58	64.44	66.53	67.50	65.92	63.82	65.07	76.65	77.52	79.25	79.35
Stay-water type	k_1	-11.57	-10.51	-13.51	-12.67	-14.53	-11.83	-12.41	-17.09	/	/	/	/
	b_1	73.97	74.92	79.40	80.73	83.65	82.99	83.92	87.60	/	/	/	/
	k_2	-57.73	-67.62	-99.96	-130.30	-115.30	-70.27	-67.64	-61.82	/	/	/	/
	b_2	113.89	140.78	208.95	272.36	242.06	156.77	145.64	128.5	/	/	/	/
	R^2	0.87**	0.94**	0.94**	0.90**	0.95**	0.91**	0.92**	0.89**	/	/	/	/
	F value	51.36	131.02	132.63	70.37	148.81	82.69	97.49	66.02	/	/	/	/
	P_1	0.86	1.15	1.50	1.63	1.57	1.26	1.12	0.92	/	/	/	/
	Water content at P_1	63.96	62.80	59.15	60.09	60.81	68.05	70.06	71.96	/	/	/	/

Note: The water dynamics of maize leaves were be fitted by three linear model, and the equations as follows:

$$\begin{cases} \text{Stage I:} \quad y = k_1 \cdot x + b_1 \,, & \mathrm{A} < x \ll B \\ \text{Stage II:} \quad y = k_2 \cdot x + b_2 \,, & B \ll x \ll C \\ \text{Stage III:} \quad y = b_0 \quad\quad , & x \gg C \end{cases}$$

Where k_1 and k_2 were the slopes of the fitting equations, and $0 > k_1 > k_2$. They represented the rate of water loss. The average value of k_1 and k_2 were -23.00 and -142.68 for General type, and were -13.01 and -83.83 for Stay-water type.

The b_1 and b_2 were the intercepts of the equations. The b_1 was related to initial water content, and the average value was about 84.76% (General type and Stay-water type were 88.61% and 80.90% respectively). The b_2 has no special physiological significance.

Point B was the turning point (P_1) from the slow loss stage (Stage I) to the rapid loss stage (Stage II), which represented the irreversible death critical point of the physiological function of the organ, and the corresponding water value was the death critical water content. For General type, the P_1 could be fitted by: $P_1 = -0.011x^2-0.08x+0.89$, $R^2=0.92$. For Stay-water type, the P_1 could be fitted by: $P_1 = -0.057x^2-0.39x+0.87$, $R^2=0.91$. The death critical water content of two types could be fitted by: $y = -1.86x+58.16$, $R^2=0.77$. The lowest critical water content was 60%. Where the x was leaf positions.

Point C was the balance critical point (P_2) between water content of organs and that in environment around, and the corresponding water value was the balance water content b_0. For General type, the P_2 could be fitted by: $P_2 = -0.012x^2-0.12x+1.16$, $R^2=0.93$. The Stage III was not observed for Stay-water type, so there was no turning point C.

The X-axis represented a time course, such as days after sowing, days after silking, or other time series. The Y-axis represented the water content of the stalk internodes or leaves. Where, the X-axis represented Normalized growth period (normalization of the days after silking).

Supplementary Table S3 Detail information of parameters of water dynamics equation of maize stalks in different types

Cultivars type	Parameters	Stalks Position														
		(0)	(-1)	(-2)	(-3)	(-4)	(-5)	(-6)	(-7)	(-8)	(-9)	(-10)	(-11)	(-12)	(-13)	(-14)
General type	k_1	/	-13.95	-13.42	-12.82	-9.81	-8.81	-9.14	-9.61	-9.61	-8.40	-8.75	-6.99	-6.15	-5.87	-5.50
	b_1	/	82.52	83.99	84.35	82.37	81.89	82.39	83.66	83.66	84.55	86.09	86.16	87.22	87.92	88.46
	k_2	-64.69	-137.00	-103.40	-127.40	-76.47	-68.01	-59.25	-50.53	-50.53	-31.00	/	/	/	/	/
	b_2	86.58	202.59	189.04	249.71	179.33	169.04	156.90	149.21	149.21	124.68	/	/	/	/	/
	b_0	8.50	9.28	9.05	16.14	/	/	/	/	/	/	/	/	/	/	/
	R^2	0.90**	0.92**	0.94**	0.86**	0.80**	0.77**	0.78**	0.79**	0.79**	0.78**	0.68**	0.59**	0.48**	0.45**	0.44**
	F value	398.11	246.77	352.11	129.06	113.90	94.89	97.08	106.28	106.28	96.47	184.02	126.42	80.87	63.29	34.87
	P_1	0.98	1.17	1.44	1.45	1.47	1.49	1.60	1.60	1.78	0.98	/	/	/	/	/
	P_2	1.20	1.41	1.74	1.83	/	/	/	/	/	/	/	/	/	/	/
	Water content at P_1	/	68.90	68.33	65.86	68.10	68.93	68.79	68.27	68.27	69.63	/	/	/	/	/
Stay-water type	k_1	/	-26.79	-11.80	-9.65	-8.85	-8.17	-6.90	-5.82	-6.67	-5.66	-9.34	-11.24	/	/	/
	b_1	/	92.60	83.30	82.42	82.85	82.77	82.18	82.19	83.86	83.80	88.14	90.79	/	/	/
	k_2	/	-75.09	-146.00	-97.79	/	/	/	/	/	/	/	/	/	/	/
	b_2	/	159.31	312.09	242.84	/	/	/	/	/		/	/	/	/	/
	R^2	/	0.80**	0.84**	0.70**	0.58**	0.58**	0.43**	0.28**	0.24**	0.24**	0.46**	0.58**	/	/	/
	F value	/	34.28	44.79	18.90	38.63	38.58	21.41	11.57	10.08	9.49	19.66	27.21	/	/	/
	P_1		1.38	1.70	1.82	/	/	/	/	/	/	/	/	/	/	/
	Water content at P_1		55.61	63.19	64.85	/	/	/	/	/		/	/	/	/	/

Note: the equation was same as Supplementary Table S2. The average value of k_1 and k_2 were -9.20 and -76.83 and they increased from top to bottom. The k_1 and k_2 could be fitted by linear equations: for k_1: $k_1 = -0.60x-13.74$, R^2=0.88, and for k_2: $k_2 = -8.40x-114.62$, R^2=0.53. Where the x was the stalk positions ($x \leq 0$).
